# Beyond Fifty Years of Millard's Rotation-Advancement Technique in Cleft Lip Closure: Are There Many “Millards”?

**DOI:** 10.1155/2012/731029

**Published:** 2012-12-06

**Authors:** Renato da Silva Freitas, Pedro Bertoco Alves, Gisele Keiko Machado Shimizu, Júlia Fortes Schuchovski, Marlon Augusto Câmara Lopes, Ivan Maluf, Antonio Jorge V. Forte, Nivaldo Alonso, Joseph Shin

**Affiliations:** ^1^Section of Plastic and Reconstructive Surgery, Federal University of Paraná, 80060-240 Curitiba, PR, Brazil; ^2^Section of Plastic and Reconstructive Surgery, Yale University School of Medicine, New Haven, CT 06510, USA; ^3^Section of Plastic and Reconstructive Surgery, São Paulo University, 01246-903 São Paulo, SP, Brazil; ^4^Section of Plastic and Reconstructive Surgery, Albert Einstein College of Medicine, Bronx, NY 10461, USA

## Abstract

In 1955, Millard developed the concept of rotation-advancement flap to treat cleft lip. Almost 6 decades later, it remains the most popular technique worldwide. Since the technique evolved and Millard published many technical variations, we decided to ask 10 experienced cleft surgeons how they would mark Millard's 7 points in two unilateral cleft lip patient photos and compared the results. In both pictures, points 1 and 2 were marked identically among surgeons. Points 3 were located adjacent to each other, but not coincident, and the largest distances between points 3 were 4.95 mm and 4.03 mm on pictures 1 and 2, respectively. Similar patterns were obtained for points 4, eight of them were adjacent, and the greatest distance between the points was 4.39 mm. Points 5 had the most divergence between the points among evaluators, which were responsible for the different shapes of the C-flap. Points 6 also had dissimilar markings, and such difference accounts for varying resection areas among evaluators. The largest distances observed were 11.66 mm and 7 mm on pictures 1 and 2, respectively. In summary, much has changed since Millard's initial procedure, but his basic principles have survived the inexorable test of time, proving that his idea has found place among the greatest concepts of modern plastic surgery.

## 1. Introduction

In 1955, Millard developed the concept of rotation-advancement flap to treat cleft lip, which became the most popular technique worldwide [1]. Many other authors published their variations of the original Millard technique [2]. The procedure consists of a lateral flap advancement into the upper lip combined with downward rotation of the medial segment, preserving the philtrum. After that, the author published 41 indexed papers and several book chapters [3–10]. In 1976, Millard published the “trilogy” entitled *Cleft Craft: The evolution of its surgery*, which eventually became instrumental in facial cleft treatment [11].

We reviewed these articles to identify the evolution of the technique described by Ralph Millard Junior [1, 3–10] and compared its variation among 10 well-known cleft surgeons.

## 2. Methods

**Figure 1:**
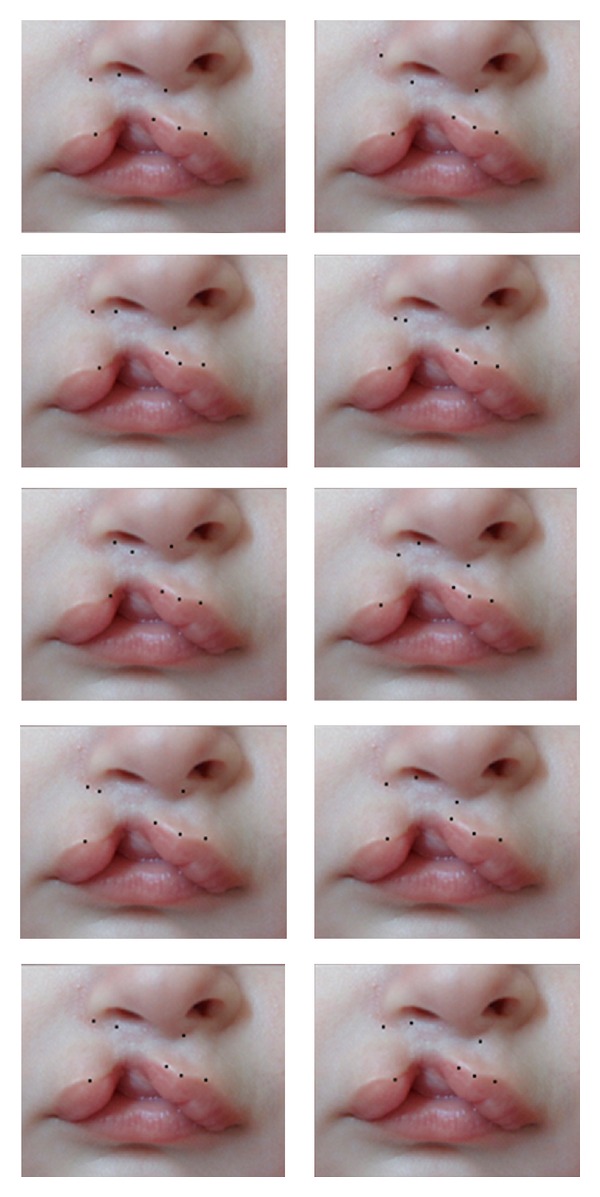
Millard's seven standard points marked by 10 respected craniofacial plastic surgeons separately on picture 1.

**Figure 2:**
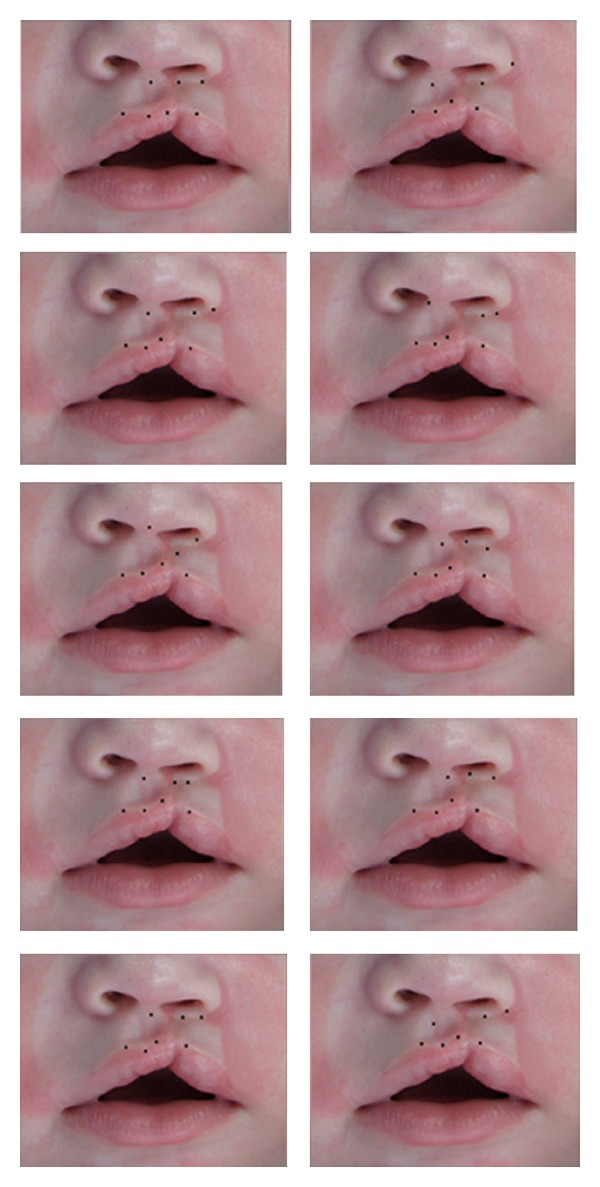
Millard's seven standard points marked by 10 respected craniofacial plastic surgeons separately on picture 2.

We carried out a systematic review of all indexed articles published by Millard since 1957. In addition, two photographs (picture 1 and picture 2) of patients with unilateral cleft lip were sent to 10 well-known Brazilian craniofacial surgeons, and we asked them to draw on them Millard's markings (Figures 1 and 2). They were asked to mark the seven standard points on the pictures and to draw the flaps afterwards. Point 1 was placed in the center of cupid bow at the vermillion border. Point 2 was in the normal side philtrum at the vermillion border. Points 3 were in the affected side philtrum at the vermillion border. Point 4 was in the mucous cutaneous border on the lateral segment of the affected side. Point 5 was at the end of the C flap. Point 6 was at the alar base and defines the height of the lateral incision. Point 7 was at the end of the lateral segment incision, around the nostril sill. Adobe Photoshop 12.0 was used to superimpose the markings on the same picture. ImageJ software (National Institutes of Health, Bethesda, MD) was employed to measure the distances between markings.

## 3. Results

It is interesting to analyze the evolution of the technique. The first diagram proposed by Millard in 1957 describes the X-Y-C flaps [1]. In 1958, he renames the flaps A-B-C and slightly modifies their designs [3]. In 1964, the B flap design changed again [6]. Then, in 1968, the B flap incision goes around the nostril sill and the back cut is introduced on the A flap [7]. In 1990, he also depicts his rhinoplasty markings on the drawing published in his paper [12]. Finally, similar markings are shown on his last published paper regarding his technique from 1998 [10]. 

**Figure 3:**
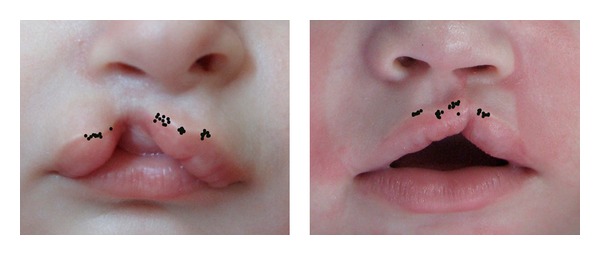
Superimposed image showing marking pattern for point 1, 2, 3, and 4 on picture 1 (left) and picture 2 (right).

In both pictures, points 1 and 2 were marked identically among surgeons. Point 3 was located adjacent to each other, but not coincident, and the largest distance between points 3 was 4.95 mm and 4.03 mm on pictures 1 and 2 respectively (Figure 3). Similar patterns were obtained for point 4, eight of them were adjacent, and the greatest distance between points was 4.39 mm. Two cases were marked very differently, one was 3.2 mm laterally and the other was 6.3 mm medially (picture1). Regarding picture 2, three evaluators had coincident markings, 3.81 mm medial to the other seven, which were also coincident.

**Figure 4:**
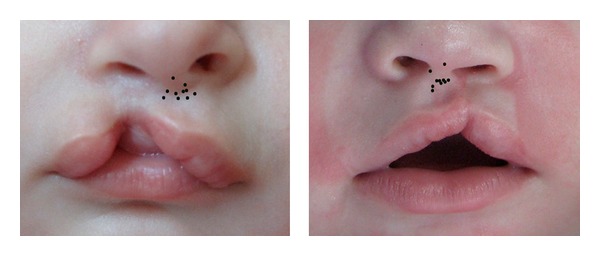
Superimposed image showing marking pattern for point 5 on picture 1 (left) and picture 2 (right).

Points 5 had the most divergence between the points among evaluators, which were responsible for the different shapes of the C flap (Figure 4). The greatest distances between these points were 11.20 mm on picture 1 and 10.86 mm on picture 2. The most contrasting points were located in one case on the columella and in another case in the medial portion of the unaffected nostril opening.

**Figure 5:**
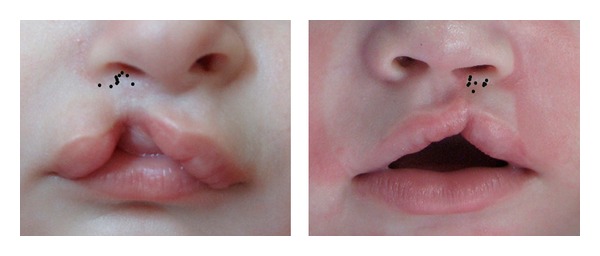
Superimposed image showing marking pattern for point 6 on picture 1 (left) and picture 2 (right).

**Figure 6:**
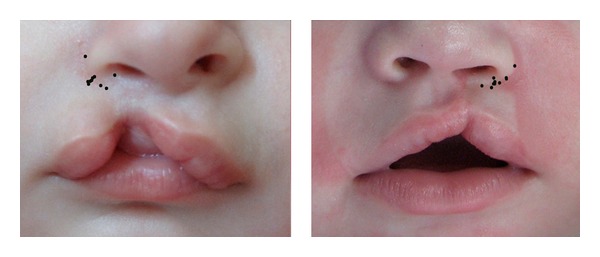
Superimposed image showing marking pattern for point 7 on picture 1 (left) and picture 2 (right).

Points 6 also had dissimilar markings, and such difference accounts for varying resection areas among evaluators. The largest distances observed were 11.66 mm and 7 mm on pictures 1 and 2, respectively (Figure 5). Regarding points 7, only one surgeon extended his marking more laterally, going around the nasal alae on both pictures. Six plastic surgeons placed coincident markings, and three set the points more medially on picture 1, but on picture 2 eight evaluators set the markings medially compared to picture 1. The largest distances between the points were 13.45 mm and 14.08 mm on pictures 1 and 2, respectively (Figure 6).

## 4. Discussion

Millard described in his first paper his well-known technique: “…why not radically free the entire medial lip element from its attachment to the nose and rotate it as a whole into its rightful position.” And he continues saying “…has the philtrum groove been preserved and the cupid's bow brought into functioning position, a small flap (c) continuous with the columella has been created which later will turn up to make the nostril sill.” When he described the lateral flap, he pointed that “the triangular gap left is to be filled by advancement of the upper portion of the lip element from the weak side. It is probably better to begin incision Y with its transverse cut just inferior to the alar base and let it curve slightly downward to facilitate the advancement. The length of this cut is a matter of judgment.” To finalize, he alerted saying “yet the last few millimeters which make all the difference must depend upon the *sculptor *and *his clay*” [1].

Later on, he described some details: (1) the rotation incision must be radical and extend just past the midline to allow adequate drop of the Cupid's bow component A; (2) advancement of flap B from the lateral lip element in severe clefts requires tension which may be hazardous in the newborn whereas at two to three months of age this tension becomes a minor concern; (3) criticism of this technique has been aimed at the long oblique scar. Actually, the scar disappears into the philtrum line; (4) approximation of the vermillion, with or without interdigitating flaps, usually calls for a minor trimming or revision after six months to perfect symmetry of the free edge of the bow; (5) medial advancement of the lateral lip flap B with lateral advancement of little flap C achieves a striking nasal correction even in severely distorted noses; and (6) absolute minimal discard of tissue [4].

After 50 years of the first rotation-advancement procedure by Millard on the lip of a small Korean boy [11], many changes happened in the original technique, including his personal changes. His first diagrams were not intelligible enough, as he previously wrote, but we added them in due to their importance. As we noticed in our study, different surgeons designed distinct flaps, all following the same technique, which brings up the point that this technique will adapt to fit the great array of possible anatomic variations within a cleft lip. More specifically, point 7, which represents the end of the lateral segment incision, around the nostril sill, has suffered the most change. A very short incision up to the medial portion of the nostril sill was proposed on 1957, which was subsequently modified in 1968 by Millard himself, comprising in a lateral extension to better address the alar rotational deformity. However, due to the poor scar quality accompanying this long incision, other craniofacial surgeons have decided reinstate the initial short incision to prevent full nostril encirclement. In fact, our study shows that only one surgeon extended his marking more laterally, while all others preferred a shorter cut. Furthermore, point 5 markings exhibit great discrepancy, which accounts for the wide C flap variation in size and design. A small or large backcut, decided upon surgeon's personal experience, aims to provide adequate advancement, which would decrease the likelihood of scar revision in the future secondary to closure under tension or tissue retraction.

Moreover, surgeons that do not obtain good results with this technique must ask themselves whether modification is required or whether a different pattern of markings needs to be done. In fact, Millard brought up the point that his technique would allow great variability of flaps and such plasticity inherent to this operation allows the experienced surgeon to shine. Nevertheless, the novice apprentice must be aware that, without proper markings, his surgery is destined to fail. In summary, much has changed since Millard's initial procedure, but his basic principles have survived the inexorable test of time, proving that his idea has found place among the greatest concepts of modern plastic surgery.
